# What is the evidence for the performance of generic preference-based measures? A systematic overview of reviews

**DOI:** 10.1007/s10198-017-0902-x

**Published:** 2017-05-30

**Authors:** Aureliano Paolo Finch, John Edward Brazier, Clara Mukuria

**Affiliations:** Health Economics and Decision Science, School of Health and Related Research, University of Sheffield, West Court, 1 Mappin Street, Sheffield, S1 4DT UK

**Keywords:** Preference based measures, Psychometric properties, Quality of life, Review, I

## Abstract

**Objective:**

To assess the evidence on the validity and responsiveness of five commonly used preference-based instruments, the EQ-5D, SF-6D, HUI3, 15D and AQoL, by undertaking a review of reviews.

**Methods:**

Four databases were investigated using a strategy refined through a highly sensitive filter for systematic reviews. References were screened and a search for grey literature was performed. Identified citations were scrutinized against pre-defined eligibility criteria and data were extracted using a customized extraction template. Evidence on known group validity, convergent validity and responsiveness was extracted and reviewed by narrative synthesis. Quality of the included reviews was assessed using a modified version of the AMSTAR checklist.

**Results:**

Thirty reviews were included, sixteen of which were of excellent or good quality. The body of evidence, covering more than 180 studies, was heavily skewed towards EQ-5D, with significantly fewer studies investigating HUI3 and SF-6D, and very few the 15D and AQoL. There was also lack of head-to-head comparisons between GPBMs and the tests reported by the reviews were often weak. Where there was evidence, EQ-5D, SF-6D, HUI3, 15D and AQoL seemed generally valid and responsive instruments, although not for all conditions. Evidence was not consistently reported across reviews.

**Conclusions:**

Although generally valid, EQ-5D, SF-6D and HUI3 suffer from some problems and perform inconsistently in some populations. The lack of head-to-head comparisons and the poor reporting impedes the comparative assessment of the performance of GPBMs. This highlights the need for large comparative studies designed to test instruments’ performance.

**Electronic supplementary material:**

The online version of this article (doi:10.1007/s10198-017-0902-x) contains supplementary material, which is available to authorized users.

## Introduction

Cost utility analysis (CUA) is increasingly used to inform health policy on whether new interventions should be made available within a healthcare system. In CUA, benefits are measured in quality adjusted life years (QALYs) using an index that combines the length of life and the health related quality of life (HRQoL) of patients [1]. HRQoL is estimated using preference-based measures (PBMs).

A limited number of generic PBMs (GPBMs) dominate the literature [2], and these are the EQ-5D, the SF-6D, the Health Utility Index mark 3 (HUI3), the Assessment of Quality of Life (AQoL) and the 15 Dimensions (15D) [3]. Their main advantage is, at least theoretically, the ability to produce values comparable across all interventions and diseases, therefore resulting in a common currency for health technology assessment. However, these instruments differ in terms of the size and content of their descriptive systems, the valuation methods and the populations used to value the health states [3], often generating substantially different utility values [4]. Differences in the size and content of the descriptive systems may limit the appropriateness of GPBMs in certain populations, while differences in the valuation methods and the populations used to value health states limits comparability between measures [1, 5, 6]. Given this variability between instruments, a key issue in the conduct and use of CUAs is the selection of instrument for measuring health state utility values. On the one side, the selected measure should be appropriate for the group of patients being examined in the evaluation in terms of its ability to detect meaningful changes; on the other side, the selected measure should ensure comparability between studies within the conditions and/or between conditions (depending on jurisdiction), to ensure an efficient allocation of resources.

To help address the selection of measures, there is a growing body of literature investigating the empirical validity (construct validity) and responsiveness of GPBMs in different populations. Validity has been defined as how well an instrument measures what it is intended to measure [7, 8], while responsiveness is a related concept on the ability of a measure to detect changes in health when these have occurred [9]. There is an increasing number of systematic reviews summarizing the validity and responsiveness of GPBMs in either a specific population or for a specific GPBM. However, it is difficult to draw conclusions regarding the performance of these measures, as the evidence is piecemeal. This study seeks to address the gap by providing a summary of the overall construct validity and responsiveness of five GPBMs, including the coverage and nature of the evidence in different conditions based on existing reviews, through an overview of reviews.

Overviews of reviews compile evidence from multiple reviews into a single accessible and usable document, offering a “friendly front end” platform for decision makers [10]. The steps required for conducting an overview of reviews are similar to those used in systematic reviews and are described in detail in Higgins and Green [10]. Broadly, these involve designing a searching strategy, screening the references obtained using a set of pre-defined eligibility criteria, assessing the reviews in terms of their quality and summarizing their evidence in an easily accessible format. The methods used in this overview of reviews are described in detail below.

## Methods

An overview of reviews was undertaken. Consistent with the Cochrane collaboration guidelines [10] all phases of this study were planned and summarized in an overview protocol (available from authors). Formal guidance on reporting of overviews of systematic reviews does not exist, but whenever possible, we followed the 27-item checklist covering important information needed in reporting systematic reviews and meta-analysis of the preferred reporting items for systematic reviews and meta-analysis (PRISMA) [11].

### Search strategy and study identification

A search strategy was developed to identify systematic reviews on the validity and responsiveness of the five most commonly used GPBMs for adults, across all disease classes. The search combined free text and controlled vocabulary words, including “quality of life”, “patient reported outcome”, “preference based instrument”, “psychometric characteristic”, “EQ-5D”, “SF-6D”, “HUI3”, “AQoL” and “15D”, all with spelling variations, acronyms and related terms (Appendix I). A highly sensitive searching filter for systematic reviews and meta-analysis developed by the information services team of the Canadian Agency for Drugs and Technologies in Health was used to refine the search [12], which was not limited by date or language restrictions. Medline, Embase, Cochrane Library and Scharr HUD electronic databases were investigated. In addition, references of the included reviews were screened and a complementary search on Google Scholar was performed.

Identified citations (both published and grey literature) were assessed against the following set of pre-defined eligibility criteria. Reports were eligible for inclusion if they were reviews, they examined construct validity or responsiveness of at least one GPBM, their main focus was on an adult population (defined as ≥18 years old) and they summarized results reporting information at the study level (either in the review text, tables or appendix). Systematic reviews were excluded if they reported results only in aggregate form, if they only examined psychometric characteristics other than construct validity or responsiveness (e.g. reliability or face validity), if they only included translations of a GPBM, if they were not in English or if they were only in a poster presentation.

### Quality assessment of the reviews

Quality was assessed using a modified eight question version of the AMSTAR checklist for systematic reviews [13] with items weighted for importance based on the research team views (See Appendix Table 2). Questions on the “comprehensiveness of the literature search”, the “presence of a quality assessment tool” and the “use of quality scores to formulate conclusions” were assigned two points as they were considered essential for the correct identification and assessment of quality of studies included in reviews. “Characteristics of the included studies” was assigned 1.5 points, as these might significantly impact on the results. “Presence of duplicate data selection and extraction” and “double blinding” (although rarely used in systematic reviews of psychometric evidence) were assigned a score of one since they strengthen the reliability of the selection process. Questions on providing an a priori design, which minimizes the chance of results being changed once searches have being completed, a list of included studies and conflicts of interest were given a weight of 0.5 as these were considered to have less of an impact on reviews of psychometric studies. Questions in AMSTAR on the “methods used to combine findings”, the “likelihood of publication bias” and the “status of publication used as an inclusion criterion” were excluded because they were considered irrelevant for systematic reviews of measures’ psychometric performance.

The resulting checklist has a minimum score of 0 and a maximum score of 10. As a way to categorize the quality of systematic reviews, arbitrary cut-offs were assigned, considering them of excellent quality if they received a score ≥7.5, of good quality if they received a score ≥5 and of poor quality with a score <5. Scores for both the original and the modified checklists are provided in the Appendix Table 3.

### Data extraction

A customized extraction template was designed and piloted on 5 reviews. Information on review characteristics (e.g. review objectives, number of studies included, disease classes investigated, condition examined) and details of the psychometric assessments undertaken were extracted. In the case of a review published in several places, then the article with the most up-to-date data was used, supplemented by additional evidence contained in the other sources. When different reviews included the same study, the most complete data for that study were extracted, supplemented by the evidence contained in the other review and presented in the results for only one of the two reviews to avoid double counting of studies.

### Assessment of findings

#### Validity

Validity of an instrument should ideally be assessed by comparing it to a gold standard measure of the construct of interest. Where a gold standard or criterion does not exist, psychometricians use indirect indicators of validity [14]. One indicator is the ability of an instrument to distinguish between groups known or thought to differ in the trait or behaviour, such as defining groups by severity of condition or patients vs general population. Care should be paid in using traits that are relevant for GPBM assessment, as not all traits used to test HRQoL are relevant for testing GPBMs (for a detailed discussion of traits relevant for GPBM assessment please see Brazier et al. [14]). Assessment of whether or not known group validity is evident can then be based on whether those with poorer health also have lower utility scores, using appropriate tests to assess whether these differences are statistically significant (e.g. *t*-tests) and important in magnitude (e.g. using standardized effect sizes (SES), which is the difference in the scores divided by the pooled standard deviation).

Another indicator is convergent validity, which examines the extent to which two measures of the same or similar concept agree with each other, for example by using correlations. The magnitude of the correlation is used to judge the extent to which GPBMs are related to the comparison measure.

#### Responsiveness

Responsiveness focuses on a measure’s ability to reflect changes that have occurred in health [9, 14], such as by comparing patients before and after a successful treatment. Change is usually assessed based on whether differences in utility scores are statistically significant and their standardized magnitudes coherent with the change that has occurred, using standardized effect sizes (SES) or standardized response means (SRMs) (i.e. the mean change divided by the standard deviation of the change scores).

#### Criteria for psychometric assessment

Criteria are required to judge whether measures meet the psychometric properties being assessed. Cohen’s criteria have been used in this overview [15]. Correlations are very strong if >0.6; strong between 0.5 and 0.6; moderate between 0.49 and 0.3; and weak if ≤0.29 [15]. Moderate to very strong correlations were taken as an indicator of convergent validity. SES and SRMs were judged as large if they were ≥0.80; moderate between 0.50 and 0.79; and small between 0.2 and 0.49 [15]. Moderate to large ESs and SRMs were taken as a sign of construct validity or responsiveness. Statistical significance was also considered as evidence to support known group validity and responsiveness. These criteria only provide indicative guidance on the psychometric characteristics of an instrument. Judgements must also be made based on the quality of studies included and the characteristics of the indirect indicators that are used.

#### Reporting

Evidence is presented in summary tables by measure and condition and reviewed by narrative synthesis. In the summary tables, symbols are used to identify where evidence supports validity or responsiveness (✓), suggests poor validity or responsiveness (✗), is mixed (±), which indicates some supporting evidence and some against, inconclusive (/), when evidence is lacking, e.g. data too sparse, or NR when the measure is not reported in the review. Conditions are grouped using the international classification of diseases [16]. AQoL 8D and 15D results are only presented in the text due to the limited evidence found.

## Results

**Fig. 1:**
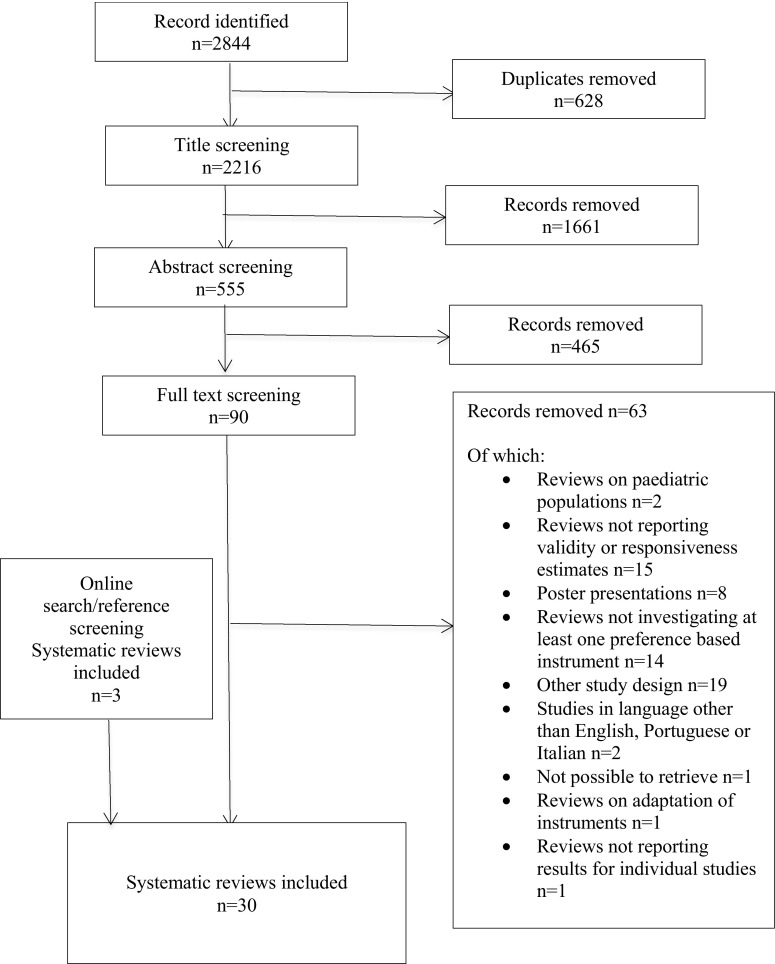
Flow diagram

A total of 2216 potentially relevant articles were identified after removing duplicates. Title and abstract screening excluded 1661 and 465 records, respectively, and full text screening excluded an additional 63. Online search and reference screening found 3 reviews that had not been detected by database searches. Consequently, 30 reviews were included [17–46]. Figure 1 summarizes the selection process. A list of included and excluded reviews is provided in Appendix Tables 4 and 5.

### Characteristics of the included reviews

The number of studies included in the reviews varied significantly,^1^ from five [38] to 122 [39]. Most reviews included a mix of randomized clinical trials (RCTs), cross-sectional, cohort and longitudinal studies, or a mix of other experimental and/or observational designs, apart from Devine et al. [38] which focused on longitudinal studies and Holloway et al. [45] which focused on RCTs. One review by Bansback et al. [43] included only economic evaluations. Table 1 summarizes the main characteristics of the included reviews.

**Table 1:** Characteristics of the included reviews

Disease area	Report	Condition/population	Quality	PBM/s included	Aims and objectives	Number of reports (number of studies)*
Autoimmune system	Castelino [34]	Systemic lupus erythematosus	Poor	EQ-5D SF-6D	To evaluate the development and psychometric properties of health related quality of life measures used in adults with systemic lupus erythematosus	13 (13)
	Holloway [42]	Systemic lupus erythematosus	Poor	EQ-5D	To create a conceptual model of the humanistic and economic burden of systemic lupus erythematosus and review the patient reported outcomes used to measure the concept in SLE clinical trials	68 (68)
Cardiovascular system	Dyer [31]	Heart disease	Good	EQ-5D SF-6D HUI3	To synthesize the evidence on the validity and reliability of the EQ-5D in studies within the cardiovascular field; to summarize the EQ-5D based score reported in studies within the cardiovascular field; and to attempt to stratify mean utility scores according to level of disease severity	66 (66)
Ear	Yang [24]	Hearing impairment	Good	EQ-5D SF-6D HUI3	To assess the reliability, validity and responsiveness of the EQ-5D, HUI3 and SF-6D for measuring health related quality of life in people with hearing impairment	18 (14)
Endocrine, nutritional and metabolic diseases	Janssen [20]	Type 2 diabetes	Good	EQ-5D SF-6D 15D	To summarize the evidence on the validity, reliability and responsiveness of the EQ-5D in studies of diabetes type 2	59 (59)
	Speight [39]	Type 2 diabetes	Poor	EQ-5D	To clarify the measurement of QoL in terms of conceptualization, terminology and psychometric properties, to review the instruments that have been most frequently used to assess QoL in diabetes and make recommendations in how to select measures appropriately	19 (19)
Eye	Tosh [23]	Visual impairment	Good	EQ-5D SF-6D HUI3	To assess the appropriateness of the EQ-5D, HUI3 and SF-6D in patients with visual disorders due to the different ways particular conditions affect HRQoL	31 (31)
Genitourinary system	Davis and Wailoo [18]	Urinary incontinence	Good	EQ-5D SF-6D 15D AQoL-8	To assess the appropriateness of the EQ-5D in people with urinary incontinence	17 (17)
	Wu [30]	HIV	Good	EQ-5D	To examine the responsiveness of two health related quality of life measures used in clinical trials involving HIV infected adults	17 (17)
Gynaecological problems	Sanghera [43]	Menorrhagia	Poor	EQ-5D	To review which economic measures have been used or assessed in menorrhagia and present criteria for deciding which measure is the most appropriate	56 (56)
Haematological problems	Szende [29]	Haemophilia	Good	EQ-5D HUI3	To review and evaluate the performance of health related quality of life and other health status measures used in studies of haemophilia in adult patients and provide recommendations for future research	19 (19)
Musculoskeletal system	Bansback [40]	Rheumatoid arthritis	Poor	EQ-5D SF-6D HUI3	To review the clinical measures used in rheumatoid arthritis economic evaluations with respect to their relevance and sensitivity to changes in survival, health related quality of life and costs	22 (22)
	DeVine [35]	Chronic low back pain	Poor	EQ-5D	To determine the correlation of patient reported pain with physical function and health related quality of life after spine surgery and the responsiveness of pain, physical function and health related quality of life measures after spine surgery (for chronic low back pain)	5 (5)
	Hill [38]	Spinal cord injury	Poor	SF-6D	To critically review quality of life instruments used in spinal cord injury	14 (14)
	Whitehurst [33]	Spinal cord injury	Good	SF-6D	To review the use of generic preference-based instruments of health-related quality of life within the context of spinal cord injury	22 (22)
Mental health	Brazier [14]	Bipolar disorder	Good	EQ-5D	To examine the validity and responsiveness of two generic preference-based measures of health (the EQ-5D and SF-6D) and two generic non-preference-based measures (the SF-36 and SF-12) in populations with bipolar disorder	22 (22)
	Papaioannou [15]	Personality disorder	Good	EQ-5D	To assess the construct validity and responsiveness of four generic health status measures in personality disorder	10 (10)
	Papaioannou [16]	Schizophrenia	Good	EQ-5D SF-6D	To assess the construct validity and responsiveness of four generic health status measures in schizophrenia	33 (33)
	Peasgood [17]	Depression/anxiety	Good	EQ-5D SF-6D HUI3	To assess the construct validity and responsiveness of EQ-5D and SF-6D measures in depression and anxiety	26 (26)
	Hounsome [32]	Dementia	Poor	EQ-5D HUI3	To review evidence relating to the application of EQ-5D in dementia research and issues concerning its use	21 (18)
Neoplasm	Longworth [22]	Cancer	Good	EQ-5D SF-6D HUI3	To assess the reliability, validity and responsiveness of the EQ-5D, HUI3 and SF-6D for measuring health related quality of life in cancer	98 (98)
	Pickard [28]	Cancer	Poor	EQ-5D	To summarize evidence on the validity and reliability of EQ-5D in cancer	34 (34)
Nervous system	Kuspinar and Mayo [21]	Multiple sclerosis	Excellent	EQ-5D SF-6D HUI3 AQoL 8	To summarize the evidence from published literature on the psychometric properties of generic utility measures in multiple sclerosis	15 (15)
Nose	Linder [37]	Acute sinusitis	Excellent	EQ-5D	To identify and compare the performance of HRQoL instruments or symptom scores for adults with acute sinusitis	29 (29)
Others	Ching [41]	Aesthetic surgery	Poor	EQ-5D	To critically review the present literature to identify the appropriate instruments to assess outcomes in aesthetic surgery	43 (not clear)
	Derrett [19]	Injuries	Poor	EQ-5D	To describe EQ-5D administration, summarize its reliability and validity and report its outcomes in injuries	44 (41)
	Haywood [36]	Older patients	Poor	EQ-5D AQoL 8	To review the evidence relating to the measurement properties of multi-item generic patient or self-assessed measures of health in older people	122 (122)
Respiratory system	Petrillo [26]	Asthma/COPD	Poor	EQ-5D	To present and discuss the empirical evidence on the validity of generic multi-attribute utility instruments within the COPD population	22 (22)
	Pickard [27]	Asthma/COPD	Good	EQ-5D SF-6D	To synthesize literature on the validity and reliability of EQ-5D use in studies of asthma and COPD, and estimate EQ-5D utility scores associated with different stages of the disease	18 (18)
Skin and subcutaneous tissues	Yang [25]	Skin condition	Good	EQ-5D	To assess the reliability, validity and responsiveness of the EQ-5D, HUI3 and SF-6D for measuring health related quality of life in skin conditions	16 (16)

* Not all the studies included in the reviews were relevant to the research question investigated in this overview. However, this overview draws on more than 150 studies included in the 30 reviews

### Quality of included reviews

Two reviews [24, 40] received an assessment of excellent quality and 14 of good quality [17–21, 23, 25–28, 30, 32, 33, 36]. The remaining 14 reviews received a poor quality assessment [22, 29, 31, 34, 35, 37–39, 41–46]. The main reason for poor quality was that reviews did not assess the quality of the included papers themselves and, consequently, did not consider scientific quality appropriately in drawing conclusions. Five reviews received an AMSTAR modified score below 3, with four of them reporting a literature search that was not considered comprehensive (i.e. terms were not derived paying attention to synonyms, acronyms and related terms for the building blocks of the research question) [29, 37, 42, 44] and none of these performed a double-blind study selection [29, 37, 42, 44, 46].

#### Breadth and depth of the evidence

Twenty-nine reviews reported information for the EQ-5D, twelve for the SF-6D, eight for the HUI3, two for the 15D and three for the AQoL 8 dimensions.

EQ-5D psychometric characteristics were presented for conditions across 16 ICD classes of disease codes (Table 2). Two reviews reported EQ-5D characteristics in a class not specified (i.e. aesthetic surgery in Ching [44] and older population in Haywood [36]). SF-6D psychometric performance was reported for conditions related to 9 classes of disease, HUI3 to 7 classes, and 15 D and AQoL only to 2 classes of disease.

**Table 2:** Main EQ-5D, SF-6D and HUI3 results

		Authors	Condition examined or population examined	Known groups			Convergent validity			Responsiveness		
				EQ-5D	SF-6D	HUI3	EQ-5D	SF-6D	HUI3	EQ-5D	SF-6D	HUI3
Disease area/population	Autoimmune system	Castelino [34]	Systemic lupus erythematosus			N/R	✓	±	N/R	✓	✓	N/R
		Holloway [42]	Systemic lupus erythematosus	✓	N/R	N/R	✓	N/R	N/R		N/R	N/R
	Cardiovascular system	Dyer [31]	Cardiovascular diseases	✓✓✓✓✓✓✓✓±✗✗✗			✓✓✓✓✗✗	✓✗	✓✗	✓✓✓✓✓✓✓✓✓✓✓✓✓✓✓✓✓✓±±±✗✗✗✗✗✗✗✗✗///		
	Ear	Yang [24]	Hearing impairment	✗		✓✓✓✓✗	✓/	✓	✓✓//	✓✗✗✗		✓✓✓✓✓
	Endocrine, nutritional and metabolic system	Janssen et al. [20]	Type 2 diabetes	✓✓✓✓✓✓✓✓✓✓✓✓✓✓✓✓✓✓✓✗✗	✓✗	N/R	✓✓✓✓✓✓±✗✗			✓✓✓✓✓✗/		N/R
		Speight [39]	Diabetes		N/R	N/R		N/R	N/R	✗	N/R	N/R
	Eye	Tosh [23]	Visual disorders	✓✓✓✓✓✓✓✓✓✓✓✓✓✓✓✓✓±±±±±±✗✗	✓	✓✓	✓✓✓✓±✗✗✗✗	✓	✓	✓±✗		✓
	Genito-urinary system	Davis and Wailoo [18]	Urinary incontinence	✓✓✓✓±	✓	N/R	✓✓✓✓✓✓✓//		N/R	✓✓✓✓✓✓✓±		N/R
		Wu [30]	HIV	✓	N/R	N/R		N/R	N/R	✓✓✗✗✗	N/R	N/R
	Gynaecological problems	Sanghera [43]	Menorrhagia	✓	N/R	N/R	✗✗	N/R	N/R	✗	N/R	N/R
	Haematological problems	Szende [29]	Haemophilia	✓✓	N/R	✓	✓	N/R			N/R	
	Musculoskeletal system	Bansback [40]	Rheumatoid arthritis				✓	✓	✓			
		De Vine [35]	Chronic low back pain		N/R	N/R	/	N/R	N/R	/	N/R	N/R
		Hill [38]	Spinal cord injury	N/R		N/R	N/R		N/R	N/R	✓	N/R
		Whitehurst [33]	Spinal cord injury		✓✓✓✗✗✗✗						✓	
	Mental health	Brazier [14]	Bipolar disorder	✓✓✗	N/R	N/R	✓✓✓✗✗	N/R	N/R		N/R	N/R
		Papaioannou [15]	Personality disorder	✓✓✗	N/R	N/R	±±	N/R	N/R	✓✓/	N/R	N/R
		Papaioannou [16]	Schizophrenia	✓		N/R	✓±±±✗✗✗✗	✗	N/R	✓✗/		N/R
		Peasgood [17]	Depression and anxiety	✓✓✓✓✓✓✓✓±✗	✓✓✓ ✓✓±	✓	✓✓✓✓✓✓	✓±	✓	✓✓✓✓✓✓✓✓✓✓✓✓✓±✗//	✓✓✓	
		Hounsome [32]	Dementia		N/R		✓✓✓✓±±✗✗	N/R	✓		N/R	
	Neoplasm	Longworth [22]	Cancer (various)	✓✓✓✓✓✓✓✓✓✓✓✓✓✓✓✓✓✓✓✓✓✓✓✓✓✗✗✗✗✗✗	✗	✓✓✓✓✓✓✓✓±✗✗	✓✓✓✓✓✓✓✓✓✓✓✓✗✗✗//	✓	✓✓✓✓✗✗/	✓✓✓✓✓✓✓✓✓✓✓✓✓✓✓✓✓✓✓✓✓✓✓✓✓✓✓✓✓±±±±±±✗✗✗✗✗///		✓✓✓✓✓✓✓✓±±
		Pickard [28]	Cancer (various)	✓✓✓✓✓✓✓✓	N/R	N/R	✓	N/R	N/R	✓✓	N/R	N/R
	Nervous system	Kuspinar and Mayo [21]	Multiple sclerosis	±±✗✗	±	✓	✓✓✓±±✗	✓✓	✓			
	Nose	Linder [37]	Rhino sinusitis		N/R	N/R		N/R	N/R	✗	N/R	N/R
	Others	Ching [41]	Aesthetic surgery		N/R	N/R		N/R	N/R	±	N/R	N/R
		Derrett [19]	Injuries	✓✓✓✓	N/R	N/R	✓✓✓✓✓±✗	N/R	N/R	✗	N/R	N/R
		Haywood [36]	Older population		N/R	N/R	/	N/R	N/R	✓	N/R	N/R
	Respiratory system	Petrillo [26]	COPD	✓±	N/R	N/R		N/R	N/R	✓✓✓	N/R	N/R
		Pickard et al. [27]	COPD and asthma	✓✓✓✓✓✓✓✓✓✓±		N/R	✓✓✓✓✓±±✗	✓	N/R	✓✗✗✗		N/R
	Skin and subcutaneous tissues	Yang [25]	Psoriasis, acne, hidradenitis suppurativa, hand eczema, venous leg ulcers	✓✓✓✓✓✓✓✓✓	N/R	N/R	✓✓✓✓✓✓✗	N/R	N/R	✓✓✓✓✓✓✓✓±±✗	N/R	N/R

Legend: ✓ results in support of validity or responsiveness; ✗ results against validity or responsiveness; ± mixed results (some tests in support and some against); / inconclusive results (e.g. data too sparse to assess correlations); N/R measure not reported in the review. Every symbol corresponds to one study. Studies reporting on more than one PBM generate more than one symbol

The amount of evidence in relation to the psychometric assessment of validity and responsiveness within conditions varied substantially, with some reviews reporting multiple psychometric analysis results and others focusing on a single type of assessment. Overall there was much less evidence available for measures other than the EQ-5D.

#### Type of evidence

##### Known groups testing

Of the 180 studies included in the systematic reviews that reported known groups validity, 77 used comparisons based on severity traits although two studies did not use all the potential severity levels [29, 34]. For the other studies comparisons were based on patients versus general population (44 studies), different types of diseases or disorders (15 studies), groups defined by an HRQoL instrument (7 studies), numbers of diseases/disorders (4 studies) and patients with or without complications (3 studies). Comparisons were also based on other groups such as discharged and not discharged patients (21 studies). Nine studies used groups that were considered inappropriate for testing GPBM validity, like age, education, different country cohorts and income. Most studies assessed known groups based on utility scores, but seven reviews [21, 24–26, 28, 30, 32] reported results for unscored dimensions of the instruments.

##### Convergent validity

Correlations with other measures were reported in 135 studies, 38 of which used a non-preference-based HRQoL measure, 32 a direct utility measure (e.g. TTO), 27 a symptom or severity measure, 18 a functional status measure, 9 another GPBM and 14 did not specify the measure used.

##### Responsiveness

Reviews reported 172 studies on GPBM responsiveness, most of which (*n* = 124) were based on comparing patients before and after a successful treatment, with 112 of these reporting statistically significant differences, 8 reporting SESs, 2 reporting SRMs and 2 not reporting the method employed. Comparisons were also based on patient groups receiving different treatments (*n* = 38; 32 reporting statistical significance and 6 reporting SESs), and patients reporting an improved health state (*n* = 6; 3 reporting SESs and 3 reporting SRMs). Four did not specify the groups used, but reported SRMs.

#### Performance of instruments by condition

The overwhelming majority of evidence in type 1 [23] and 2 [23, 42] diabetes mellitus showed that EQ-5D possessed good discrimination between severity groups, correlated moderately to strongly with other HRQoL instruments and reported changes consistent with expectations after patients’ treatment. Little evidence was found for the SF-6D, and this was mixed.

The review on diseases of the skin and subcutaneous tissues [28] (including psoriasis, acne, eczema and leg ulcers) presented results supporting EQ-5D validity and responsiveness, with only 2 out of 27 studies reporting evidence against the measure’s validity, which were weak correlations and lower SRMs for EQ-5D compared to other measures.

Two systematic reviews investigated COPD and asthma [29, 31], suggesting that the EQ-5D is generally valid based on known group comparisons of severity and patients/general population groups and correlations between the EQ-5D and non-preference-based HRQoL measures. Results for responsiveness were mixed, with two studies reporting weak SRMs of the measure, one study strong SRMs and four showing changes in the expected direction using SESs and statistical significance. The only comparative study across GPBMs reported poor correlations between EQ-5D and SF-6D.

One review each investigated the performance of the EQ-5D in urinary incontinence [21] and HIV [33]. There was evidence of validity and responsiveness in urinary incontinence [21] with five studies supporting discriminative validity based on severity levels and type of urinary incontinence, seven reporting moderate to strong correlations with HRQoL and symptom and severity measures, and five showing differences in health status from baseline to follow-up and between treatment arms. Two studies reported mixed results, one showing that the EQ-5D distinguished between some types of urinary incontinences but not others, and the other that the EQ-5D detected treatment differences only for some groups of patients, where other measures registered changes for all treatment groups. Two studies had inconclusive results for convergent validity as they did not specify the strength of correlations between measures. One study reported results for other GPBMs, supporting SF-6D, 15D and AQoL known group validity based on the assessment of severity traits. In HIV [33] responsiveness of the EQ-5D was weak, showing generally small before and after treatment SESs in the presence of moderate or large ESs for the comparator measures. The only study investigating construct validity reported a good ability of the measure to discriminate between known groups.

The EQ-5D appeared generally valid and responsive in a number of cancers [25, 31] (including lung, breast, cervical, colon, kidney, liver cancer and leukemia) although limitations were found in some studies. Twenty-five of the 31 studies examining known group differences showed that EQ-5D distinguished between cancer severities, patients/general population and groups with different types of cancer; 12 of the 17 studies examining convergent validity reported moderate to strong correlations with direct utility measures, HRQoL measures and functional status measures; and 29 of 43 studies examining responsiveness showed that the measure detected changes between treatment arms and from baseline to follow-up that were consistent with those of comparator measures. A significant amount of evidence supported HUI3 psychometric characteristics [25, 31] with 8 studies out of 11 showing good discriminative ability in distinguishing between severity levels, type of cancer and cancer patients/general population, 4 studies out of 7 reporting good convergence with functional status measures and 8 studies out of 10 a good ability to detect changes from baseline and between treatment arms. Only two studies reported information for the SF-6D. In one, the measure was not able to detect differences between cancer patients and the general population. In another, the measure correlated appropriately with a cancer HRQoL questionnaire. Very few comparative studies were reported between the investigated GPBMs, and these do not clarify which performs better.

The EQ-5D showed a mixed performance in cardiovascular diseases [34] (including coronary heart disease, cerebrovascular disease, hypertension and heart failure). Although many studies supported the instrument’s convergent validity with other GPBMs, HRQoL measures and functional status measures, and its ability to distinguish known groups based on severities of the conditions and type of conditions, two studies showed poor correlations with HRQoL measures, three had problems in distinguishing between patients and the general population, eight failed to detect statistically significant changes at follow-up and one failed to show differences between treatment arms. Three comparative studies were reported between the EQ-5D and SF-6D, the EQ-5D and HUI3, and the EQ-5D, SF-6D and HUI3. In two of them, correlation between the EQ-5D and SF-6D, and between the EQ-5D, HUI3 and SF 36 were generally poor. The third comparative study presented moderate to strong correlations between the three instruments.

The EQ-5D performance in visual disorders [26] (including macular degeneration, glaucoma, conjunctivitis, diabetic retinopathy and others) was generally mixed. Known groups showed generally poor or mixed validity using severity groups, and generally good validity using patients versus general population groups. Mixed evidence was also reported for convergent validity, with the instrument correlating moderately to strongly with clinical measures only in four of the nine studies that investigated the property. There was mixed and limited evidence for EQ-5D responsiveness, with one study reporting in support, one against and one with mixed evidence for the measure’s responsiveness. All these studies used tests of statistical significance before and after treatment. The HUI3 appeared to be valid although the evidence was limited. Two studies reported a good ability of the measure to distinguish known groups based on the severity of the condition and on patients/general population. Another study reported moderate to strong correlations with functional status measures. A fourth study showed that the HUI3 was able to detect statistically significant changes between treatment arms [26]. Only two studies reported on the SF-6D characteristics, and these showed that the measure performed better than the EQ-5D [26].

EQ-5D performance has been reviewed in only one condition of the nervous system [24], multiple sclerosis, with three studies supporting the instrument’s convergent validity and three reporting weak to moderate correlations with other HRQoL measures. Substantial evidence against the instrument’s ability to distinguish between severity groups was found, with two studies reporting that the measure distinguished only between some severity levels but not others (mixed evidence), and two showing the measure was not able to detect health status differences in any of the severity levels. Evidence for the SF-6D, HUI3 and AQoL was limited, but in support of the measure’s performance [24], with two studies reporting moderate to strong convergence of the SF-6D with HRQoL measures, two showing good discriminative ability of the HUI3 between severity groups, strong correlations of the measure with other HRQoL instruments and two showing good discriminative ability of the AQoL, with the assessment being based on severity levels.

The EQ-5D performance in hearing impairments [27] was poor, with only two studies out of the seven supporting validity and responsiveness, one reporting moderate to strong correlations with other GPBMs and the other reporting statistically significant changes of score before and after treatment. The HUI3 showed a better performance, with all known group assessments but one in favour of the instrument’s validity (based on severity traits) and most of the responsiveness tests showing an ability to detect changes in health status before and after treatment [27]. Although few comparative studies were found, all these suggested that the HUI3 performs better than the EQ-5D in hearing impairment.

Five reviews investigated the performance of the EQ-5D in mental health [17–20, 35], and all but the one on depression and anxiety showed that the instrument suffered from problems. Three studies showed low correlations between the EQ-5D and HRQoL measures in dementia; four had low correlations between the EQ-5D and the time trade-off, standard gamble and symptom specific measures in schizophrenia; two had low correlations between the EQ-5D and other measures (not specified) in bipolar disorder; and two had low to moderate correlations between the EQ-5D and symptom and severity measures in personality disorders. Evidence against the measure’s validity was also found for known groups in personality disorders and bipolar disorder, with one study showing poor discrimination between groups based on different types of personality disorders, and another poor discrimination between severity levels of bipolar disorder. Convergent validity, known groups and responsiveness results for the SF-6D and HUI3 supported the instruments’ psychometric characteristics, with the exception of an SF-6D known group test that showed mixed results in depression (discriminating only between some groups but not others) [20], although the evidence base was smaller.

Four systematic reviews reported evidence on EQ-5D and SF-6D psychometric characteristics in musculoskeletal diseases [36, 38, 41, 43]. One study reported good convergence for the EQ-5D with another HRQoL measure in rheumatoid arthritis, while another had inconclusive results in chronic low back pain, with data being too sparse to assess correlations. The SF-6D was seen to have moderate to strong convergence with an HRQoL measure in rheumatoid arthritis, but mixed known group results in spinal cord injuries, with three studies supporting the instrument’s discriminative ability and four reporting against it [36].

Evidence for the other ICD disease classes was very sparsely investigated, including haematological, gynaecological and autoimmune diseases, and diseases of the nose. Three reviews investigated injuries, aesthetic surgery and older populations, but evidence was extremely limited, although the few studies available were generally in support of the GPBMs’ psychometric characteristics [21, 31, 36, 38, 39, 43–45].

## Discussion

The aim of this overview of reviews was to summarize the evidence on the construct validity and responsiveness of five GPBMs, the EQ-5D, SF-6D, HUI3, AQoL and 15D in terms of the size, quality and nature of the evidence across different conditions, and to determine whether it is possible to draw conclusions about their relative performance. A systematic overview of reviews was undertaken that yielded 30 systematic reviews, which included more than 180 studies.

### Size and coverage of the evidence

The body of evidence was heavily skewed towards EQ-5D, with significantly fewer systematic reviews investigating HUI3 and SF-6D, and almost none investigating 15D and AQoL. Furthermore, the number of conditions covered was limited, even for EQ-5D. There were also limitations in the psychometric assessment that was reported. For example, some studies only reported convergent validity, or reported comparisons with only one other indicator. This limits the conclusions that can be drawn from the evidence, particularly in terms of comparability between different GPBMs.

### Quality, nature and reporting of evidence

Many of the reviews received an AMSTAR modified score of poor quality mainly because they did not assess the quality of the studies they included and consequently the impact of this on their synthesized results. In the presence of discordant results between studies, quality assessment can help in the interpretation and synthesis of evidence, for example by giving greater weight to more robust reports.

Reviews reported different types of evidence for each of the two indicators of validity and responsiveness, such as known groups being defined by severity, number of diseases/disorders or patient versus general population, and treated them as equally informative. Although this is common practice in empirical studies, some tests should be considered more appropriate than others. For example, the trait severity of a disease may be considered more informative than the trait number of disease/disorders in known group assessments, since the ranking of preferences might be ambiguous in the latter case, e.g. one severe condition might be worse than two mild ones. Comparing patient and general population scores is likely to be very crude. Furthermore, the tests often rely on clinical assessments that may not reflect the HRQoL of patients or preferences for the states. These aspects need more careful consideration in the phases of review analysis and synthesis, as well as for the design of primary studies.

A number of concerns exist about the way in which evidence was reported by the included reviews. Few reviews stated with clarity which thresholds were adopted in analyzing and summarizing results, making the interpretation of the definitions “strong”, “moderate” or “weak” more difficult. It was also common to find outcomes defined as “significant”, and doubts remained as to whether “significant” meant statistically significant or significant in size, or both. Some known group tests based on severity of the condition reported only part of the range of possible severity levels. This significantly weakens the value of the evidence produced.

### Performance of instruments

Despite the lack of evidence and standardization across the reviews or studies included in psychometric assessment, some broad conclusions can be drawn from this overview of reviews. Where evidence was available, it often supported the GPBM’s performance. EQ-5D appeared valid and responsive in conditions of the skin, respiratory, genitourinary, endocrine, nutritional and metabolic diseases, and for the majority of cancers where there was evidence; SF-6D was found to be valid and responsive in mental health and in diseases of the eye, the nervous and the genitourinary systems; HUI3 showed good validity and responsiveness in cancer, diseases of the eye, the ear, the nervous system and mental health; AQoL presented good psychometric characteristics in musculoskeletal and genitourinary conditions, and 15D in genitourinary, diabetes, nutritional and metabolic diseases. However, any attempt to compare the instruments is limited by a lack of head-to-head comparisons and the little evidence available on all GPBMs except EQ-5D.

There was also evidence of lack of appropriateness of GPBMs in some conditions. EQ-5D was found to perform poorly in hearing impairments, multiple sclerosis, personality disorders, schizophrenia and dementia, and reported mixed results in visual disorders and in some cancers. SF-6D showed inconsistencies in its ability to converge with other measures in cardiovascular and respiratory diseases and to discriminate between groups in neoplasms, while HUI3 reported mixed results for some subpopulations of neoplasms.

Most of the evidence that was used in the reviews relied on studies that used existing datasets, but this provides limited answers when investigating GPBMs’ comparative performance and it highlights the importance of designing bespoke comparative studies for this purpose. There are a few examples of these, including two large and two smaller studies where five instruments were investigated [47], and the more recent Multi Instrument Comparison (MIC) project [48], that compared a number of GPBMs and other measures across different conditions. This evidence shows that convergence between GPBMs is generally moderate to large, but that differences in scores are mostly driven by the different constructs covered by the their descriptive systems. The presence or absence of dimensions covering constructs relevant to a specific condition/disease area might serve as an explanation for the lack of validity and responsiveness noticed in some disease areas for the investigated GPBMs.

### Limitations of the overview

This overview of reviews has some important limitations. Psychometric properties of GPBMs in some conditions may have been missed because of the lack of a systematic review for those conditions. In addition, this overview has been limited by the poor reporting of some reviews/studies. This overview focused on the five most widely used generic GPBMs, but there are other methods for obtaining health state utility values which were not covered, such as condition specific PBMs, bespoke vignettes or direct valuations of patients’ health states [1]. These alternatives may provide an important source of evidence for reimbursement decisions, particularly where the existing generic measures do not appear to perform well. However, these have not been included in the current overview of reviews as GPBMs are the preferred option for CUA of health care interventions.

## Conclusions

Whenever evidence is available, it often supports the performance of GPBMs. However, the breadth and depth of this evidence is inconsistent between ICD disease classes, conditions, instruments and type of assessment. Indeed there is often no evidence at all, or what is available is severely limited in nature and quality, and rarely enables direct comparisons across measures. This highlights the need for large comparative studies designed to test the performance of instruments, therefore producing evidence that is equivalent in breadth, depth and quality for all GPBMs. In addition, more rigorous reporting of GPBM psychometric studies and reviews is recommended.

## Electronic supplementary material

Below is the link to the electronic supplementary material.

Supplementary material 1 (DOCX 128 kb)

## Footnotes

1 Not all the studies included in the reviews were relevant to the research question investigated in this overview. However, this overview draws on more than 180 studies included in the 30 reviews.
