# Supplementary material for: What is the evidence for the performance of generic preference-based measures? A systematic overview of reviews
Source: Eur J Health Econ. 2017 May 30;19(4):557–70. doi: 10.1007/s10198-017-0902-x (PMC5913394; doi:10.1007/s10198-017-0902-x)
Supplement: Supplementary file 1 — Supplementary material 1 (DOCX 128 kb) [file 10198_2017_902_MOESM1_ESM.docx]

**Appendixes**

**Table I – Searching strategy for Medline**

| *Search* | *Searching terms* |
| --- | --- |
| #1 | validity OR responsiveness OR “psychometric characteristic$” OR “psychometric aspect$” OR “psychometric propert$” |
| #2 | “Preference based instrument$” OR “preference based measure$” OR “multi-attribute utility instrument$” OR “generic adj instrument$” OR “multi adj instrument$” OR “patient reported outcome$” OR “PROMS” OR “PROM” |
| #3 | euroqol OR “euro qol” OR “eq5d” OR “eq 5d” OR “eq-5d” OR “euro adj qol” OR “eur adj qual” OR “eq adj 5d[tw]” |
| #4 | sf6D OR “sf 6D” OR “short form 6D” OR “shortform 6D” OR “sf six D” OR “sfsixD” OR “shortform six D” OR “short form sixD” OR “sf-6d” OR 6d OR 6-d OR “6 dimension[tw]” |
| #5 | “hui3” OR “hui 3” OR “health utilities index mark 3” OR “health utilities mark three” OR “hui III” OR “hui mk III” OR huiIII[tw] |
| #6 | “15D instrument” OR “fifteen D instrument” OR “fifteen dimension instrument” OR “15 dimension instrument” OR “15dimension instrument” |
| #7 | “Assessment of Quality of Life” OR AQOL OR “Assessment-of-Quality-of-Life” |
| #8 | “Quality of life” |
| #9 | systematic[sb] OR meta-analysis[pt] OR meta-analysis as topic[mh] OR meta-analysis[mh] OR meta analy*[tw] OR metanaly*[tw] OR metaanaly*[tw] OR met analy*[tw] OR integrative research[tiab]  OR integrative review*[tiab] OR integrative overview*[tiab] OR research integration*[tiab] OR research overview*[tiab] OR collaborative review*[tiab] OR collaborative overview*[tiab] OR systematic review*[tiab] OR technology assessment*[tiab] OR technology overview*[tiab] OR "Technology Assessment, Biomedical"[mh] OR HTA[tiab] OR HTAs[tiab] OR comparative efficacy[tiab] OR comparative effectiveness[tiab] OR outcomes research[tiab] OR indirect comparison*[tiab] OR ((indirect treatment[tiab] OR mixed-treatment[tiab]) AND comparison*[tiab]) OR Embase*[tiab] OR Cinahl*[tiab] OR systematic overview*[tiab] OR methodological overview*[tiab]  OR methodologic overview*[tiab]  OR methodological review*[tiab]  OR methodologic review*[tiab] OR quantitative review*[tiab] OR  quantitative overview*[tiab] OR quantitative synthes*[tiab] OR pooled analy*[tiab] OR Cochrane[tiab] OR Medline[tiab] OR Pubmed[tiab] OR Medlars[tiab] OR handsearch*[tiab] OR hand search*[tiab] OR meta-regression*[tiab] OR metaregression*[tiab] OR data synthes*[tiab] OR data extraction[tiab] OR data abstraction*[tiab] OR mantel haenszel[tiab] OR peto[tiab] OR der-simonian[tiab] OR dersimonian[tiab] OR fixed effect*[tiab] OR "Cochrane Database Syst Rev"[Journal:__jrid21711] OR "health technology assessment winchester, england"[Journal] OR  "Evid Rep Technol Assess (Full Rep)"[Journal] OR "Evid Rep Technol Assess (Summ)"[Journal] OR "Int J Technol Assess Health Care"[Journal] OR "GMS Health Technol Assess"[Journal] OR "Health Technol Assess (Rockv)"[Journal] OR "Health Technol Assess Rep"[Journal] |
| #10 | #2 OR #3 OR #4 OR #5 OR #6 OR #7 OR #8 |
| #11 | #1 AND #10 |
| #12 | #9 AND #11 |

**Table II -AMSTAR Modified version**

| **Question** | **Score** |
| --- | --- |
| Was an apriori design provided? | 0.5 points |
| Was there duplicate study selection and data extraction? | 1 point |
| Was a comprehensive literature search performed? | 2 points |
| Was a list of included studies provided? | 0.5 points |
| Where the characteristics of the included studies provided? | 1.5 points |
| Was the scientific quality of the included studies assessed and documented? | 2 points |
| Was the scientific quality of the included studies used appropriately in formulating conclusions? | 2 points |
| Was the conflict of interest included? | 0.5 points |

Minimum score 0

Maximum score 10

**Table III - Scores for original and modified AMSTAR checklist**

| *Disease area* | *Report* | *AMSTAR modified score* | *AMSTAR original score* |
| --- | --- | --- | --- |
| Autoimmune system | Castelino (34) | 2,5 | 4/11 |
|  | Holloway (42) | 3,5 | 4/11 |
| Cardiovascular system | Dyer (31) | 4,5 | 5/11 |
| Ear | Yang (24) | 7 | 7/11 |
| Endocrine, nutritional and metabolic diseases | Janssen (20) | 5,5 | 7/11 |
|  | Speight (39) | 0,5 | 2/11 |
| Eye | Tosh (23) | 7 | 7/11 |
| Genitourinary system | Davis and Wailoo (18) | 5,5 | 6/11 |
|  | Wu (30) | 5,5 | 6/11 |
| Gynaecological problems | Sanghera (43) | 2,5 | 3/11 |
| Haematological problems | Szende (29) | 4,5 | 6/11 |
| Musculoskeletal system | Bansback (40) | 4,5 | 5/11 |
|  | DeVine (35) | 3 | 4/11 |
|  | Hill (38) | 4,5 | 5/11 |
|  | Whitehurst (33) | 5,5 | 6/11 |
| Mental health | Brazier (14) | 5 | 5/11 |
|  | Papaioannou (15) | 5 | 6/11 |
|  | Papaioannou (16) | 7 | 7/11 |
|  | Peasgood (17) | 6,5 | 6/11 |
|  | Hounsome (32) | 4 | 5/11 |
| Neoplasm | Longworth (22) | 7 | 7/11 |
|  | Pickard (28) | 4 | 5/11 |
| Nervous system | Kuspinar and Mayo (21) | 8,5 | 8/11 |
| Nose | Linder (37) | 9,5 | 8/11 |
| Others | Ching (41) | 0 | 1/11 |
|  | Derrett (19) | 3 | 4/11 |
|  | Haywood (36) | 3 | 3/11 |
| Respiratory system | Petrillo (26) | 2,5 | 4/11 |
|  | Pickard (27) | 5,5 | 8/11 |
| Skin and subcutaneous tissues | Yang (25) | 7 | 7/11 |

**Table IV– List of included studies**

| **Studies** |
| --- |
| Bansback N, Ara R, Karnon J, Anis A. Economic evaluations in rheumatoid arthritis: a critical review of measures used to define health States. Pharmacoeconomics 2008;26(5):395-408.  Brazier J, Connell J, Papaioannou D, Mukuria C, Mulhern B, Peasgood T, et al. A systematic review, psychometric analysis and qualitative assessment of generic preference-based measures of health in mental health populations and the estimation of mapping functions from widely used specific measures. Health Technol Assess 2014 May;18(34):vii-viii, xiii-xxv, 1-188.  Castelino,M., Abbott,J., McElhone,K., Teh,L.S. Comparison of the psychometric properties of health-related quality of life measures used in adults with systemic lupus erythematosus: a review of the literature. Rheumatology ;52:684.  Ching S, Thoma A, McCabe RE, Antony MM. Measuring outcomes in aesthetic surgery: a comprehensive review of the literature. Plast Reconstr Surg 2003 Jan;111(1):469-80; discussion 481-2.  Davis S, Wailoo A. A review of the psychometric performance of the EQ-5D in people with urinary incontinence. Health Qual Life Outcomes 2013 Feb 18;11:20-7525-11-20.  Derrett S, Black J, Herbison GP. Outcome after injury-a systematic literature search of studies using the EQ-5D. J Trauma 2009 Oct;67(4):883-890.  DeVine J, Norvell DC, Ecker E, Fourney DR, Vaccaro A, Wang J, et al. Evaluating the correlation and responsiveness of patient-reported pain with function and quality-of-life outcomes after spine surgery. Spine (Phila Pa 1976) 2011 Oct 1;36(21 Suppl):S69-74.  Dyer MTD, Goldsmith KA, Sharples LS, Buxton MJ. A review of health utilities using the EQ5D in studies of cardiovascular disease. Health and Quality of Life Outcomes. 2010  Haywood KL, Garratt AM, Fitzpatrick R. Quality of life in older people: a structured review of generic self-assessed health instruments. Qual Life Res 2005 Sep;14(7):1651-1668.  Hill MR, Noonan VK, Sakakibara BM, Miller WC, SCIRE Research Team. Quality of life instruments and definitions in individuals with spinal cord injury: a systematic review. Spinal Cord 2010 Jun;48(6):438-450.  Holloway L, Humphrey L, Heron L, Pilling C, Kitchen H, Hojbjerre L, et al. Patient-reported outcome measures for systemic lupus erythematosus clinical trials: a review of content validity, face validity and psychometric performance. Health Qual Life Outcomes 2014 Jul 22;12:116-014-0116-1.  Hounsome N, Orrell M, Edwards RT. EQ-5D as a quality of life measure in people with dementia and their carers: evidence and key issues. Value Health 2011 Mar-Apr;14(2):390-399.  Janssen MF, Lubetkin EI, Sekhobo JP, Pickard AS. The use of the EQ-5D preference-based health status measure in adults with Type 2 diabetes mellitus. Diabet Med 2011 Apr;28(4):395-413.  Kuspinar A, Mayo NE. A review of the psychometric properties of generic utility measures in multiple sclerosis. Pharmacoeconomics 2014 Aug;32(8):759-773.  Linder JA, Singer DE, Ancker M, Atlas SJ. Measures of health-related quality of life for adults with acute sinusitis. A systematic review. J Gen Intern Med 2003 May;18(5):390-401.  Longworth L, Yang Y, Young T, Mulhern B, Hernandez Alava M, Mukuria C, et al. Use of generic and condition-specific measures of health-related quality of life in NICE decision-making: a systematic review, statistical modelling and survey. Health Technol Assess 2014 Feb;18(9):1-224.  Papaioannou D, Brazier J, Parry G. How to measure quality of life for cost effectiveness analysis in personality disorder? A systematic review. HEDS discussion paper 13/02. 2013  Papaioannou D, Brazier J, Parry G. How valid and responsive are generic health status measures, such as EQ-5D and SF-36, in schizophrenia? A systematic review. Value Health 2011 Sep-Oct;14(6):907-920.  Peasgood T, Brazier J, Papaioannou D. A systematic review of the validity and responsiveness of EQ5D and SF6D for depression and anxiety. HEDS discussion paper 12/15 (unpublished). 2012  Petrillo J, van Nooten F, Jones P, Rutten-van Molken M. Utility estimation in chronic obstructive pulmonary disease: a preference for change? Pharmacoeconomics 2011 Nov;29(11):917-932.  Pickard AS, Wilke CT, Lin HW, Lloyd A. Health utilities using the EQ-5D in studies of cancer. Pharmacoeconomics 2007;25(5):365-384.  Pickard AS, Wilke C, Jung E, Patel S, Stavem K, Lee TA. Use of a preference-based measure of health (EQ-5D) in COPD and asthma. Respir Med 2008 Apr;102(4):519-536.  Sanghera S, Frew E, Kai J, Gupta J, Elizabeth Roberts T. An assessment of economic measures used in menorrhagia: a systematic review. Soc Sci Med 2013 Dec;98:149-153.  Speight J, Reaney MD, Barnard KD. Not all roads lead to Rome-a review of quality of life measurement in adults with diabetes. Diabet Med 2009 Apr;26(4):315-327.  Szende A, Schramm W, Flood E, Larson P, Gorina E, Rentz AM, et al. Health-related quality of life assessment in adult haemophilia patients: a systematic review and evaluation of instruments. Haemophilia 2003 Nov;9(6):678-687.  Tosh,J, Brazier,J., Evans,P., Longworth,L. A review of generic preference-based measures of health-related quality of life in visual disorders. Value In Health ;15:118.  Whitehurst DG, Noonan VK, Dvorak MF, Bryan S. A review of preference-based health-related quality of life questionnaires in spinal cord injury research. Spinal Cord 2012 Sep;50(9):646-654.  Wu AW, Hanson KA, Harding G, Haider S, Tawadrous M, Khachatryan A, et al. Responsiveness of the MOS-HIV and EQ-5D in HIV-infected adults receiving antiretroviral therapies. Health Qual Life Outcomes 2013 Mar 12;11:42-7525-11-42.  Yang,Y, Longworth,L., Brazier,J. An assessment of validity and responsiveness of generic measures of health-related quality of life in hearing impairment. Quality of Life Research .  Yang Y, Brazier J, Longworth L. EQ5D in skin conditions: an assessment of validity and responsiveness. European Journal of Health Economics. 2014 |

**Appendix Table V – List of excluded studies**

| **Studies** |
| --- |
| Ades AE, Lu G, Madan JJ. Which health-related quality-of-life outcome when planning randomized trials: Disease-specific or generic, or both? A common factor model. Value in Health 2013 January-February 2013;16(1):185-194.  Adobor,R.D., Rimeslatten,S., Keller,A., Brox,J.I. Repeatability, reliability, and concurrent validity of the scoliosis research society-22 questionnaire and EuroQol in patients with adolescent idiopathic scoliosis. Spine ;35:206.  Al Sayah F, Ishaque S, Lau D, Johnson JA. Health related quality of life measures in Arabic speaking populations: a systematic review on cross-cultural adaptation and measurement properties. Qual Life Res 2013 Feb;22(1):213-229.  Alsanafi S, Werth VP. Evaluating quality of life in dermatomyositis. Expert Review of Dermatology 2010 December 2010;5(6):605-609.  Angst F, Stucki G, Aeschlimann A. Quality of life assessment in osteoarthritis. Expert Review of Pharmacoeconomics and Outcomes Research 2003 October 2003;3(5):623-636.  Arrington R, Cofrancesco J, Wu AW. Questionnaires to measure sexual quality of life. Qual Life Res 2004 Dec;13(10):1643-1658.  Ashcroft DM, Li Wan Po A, Williams HC, Griffiths CE. Quality of life measures in psoriasis: a critical appraisal of their quality. J Clin Pharm Ther 1998 Oct;23(5):391-398.  Au Eong,K.G., Chan,E.W., Luo,N., Wong,S.H., Tan,N.W., Lim,T.H., Wagle,A.M. Validity of EuroQOL-5D, time trade-off, and standard gamble for age-related macular degeneration in the Singapore population. Eye ;26:379.  Augestad LA, Rand-Hendriksen K, Kristiansen IS, Stavem K. Learning effects in time trade-off based valuation of EQ-5D health states. Value in Health 2012 March-April 2012;15(2):340-345.  Augustin M, Langenbruch AK, Gutknecht M, Radtke MA, Blome C. Quality of Life Measures for Dermatology: Definition, Evaluation, and Interpretation. Current Dermatology Reports 2012 September 2012;1(3):148-159.  Augustin M, Langenbruch AK, Herberger K, Baade K, Goepel L, Blome C. Quality of life measurement in chronic wounds and inflammatory skin diseases: Definitions, standards and instruments. Wound Medicine 2014 June 2014;5:29-38.  Balioussis C, Hitzig SL, Flett H, Noreau L, Craven BC. Identifying and classifying quality of life tools for assessing spasticity after spinal cord injury. Topics in Spinal Cord Injury Rehabilitation 2014 01 May 2014;20(3):208-224.  Barton,G.R., Sach,T.H., Avery,A.J., Doherty,M., Jenkinson,C., Muir,K.R. Comparing the performance of the EQ-5D and SF-6D when measuring the benefits of alleviating knee pain. Cost Effectiveness and Resource Allocation ;7:12.  Beresniak A, Russell AS, Haraoui B, Bessette L, Bombardier C, Duru G. Advantages and limitations of utility assessment methods in rheumatoid arthritis. J Rheumatol 2007 Nov;34(11):2193-2200.  Blome,C., Beikert,F.C., Rustenbach,S.J., Augustin,M. Mapping DLQI on EQ-5D in psoriasis: transformation of skin-specific health-related quality of life into utilities. Arch Dermatol Res ;305:197.  Boling W, Fouladi RT, Basen-Engquist K. Health-related quality of life in gynecological oncology: instruments and psychometric properties. Int J Gynecol Cancer 2003 Jan-Feb;13(1):5-14.  Brazier JE, Connell J, O'Cathain A. DO EQ-5D and SF-6D ask the right questions in mental health? A content validation using interviews with patients. Value in Health 2014 May 2014;17(3):A194.  Brazier JE, Green C, Kanis JA. A systematic review of health state utility values for osteoporosis-related conditions. Osteoporosis Int 2002 01 Oct 2002;13(10):768-776.  Brazier JE, Rowen D, Mavranezouli I, Tsuchiya A, Young T, Yang Y, et al. Developing and testing methods for deriving preference-based measures of health from condition-specific measures (and other patient-based measures of outcome). Health Technol Assess 2012 Jul;16(32):1-114.  Brazier JE, Yang Y, Tsuchiya A, Rowen DL. A review of studies mapping (or cross walking) non-preference based measures of health to generic preference-based measures. Eur J Health Econ 2010 Apr;11(2):215-225.  Brennan DS. Oral Health Impact Profile, EuroQol, and Assessment of Quality of Life instruments as quality of life and health-utility measures of oral health. Eur J Oral Sci ;121:188.  Buitinga,L, Braakman-Jansen,LM, Taal,E, Kievit,W, Visser,H, van Riel,PL, van,de Laar. Comparative responsiveness of the EuroQol-5D and Short Form 6D to improvement in patients with rheumatoid arthritis treated with tumor necrosis factor blockers: results of the Dutch Rheumatoid Arthritis Monitoring registry. Arthritis Rheum ;64:826.  Chopra I, Kamal KM. A systematic review of quality of life instruments in long-term breast cancer survivors. Health Qual Life Outcomes 2012 Jan 31;10:14-7525-10-14.  Chow MY, Morrow AM, Cooper Robbins SC, Leask J. Condition-specific quality of life questionnaires for caregivers of children with pediatric conditions: a systematic review. Qual Life Res 2013 Oct;22(8):2183-2200.  Cleland J, Gillani R, Bienen EJ, Sadosky A. Assessing dimensionality and responsiveness of outcomes measures for patients with low back pain. Pain Pract 2011 Jan-Feb;11(1):57-69.  Connell J, O'Cathain A, Brazier J. Measuring quality of life in mental health: Are we asking the right questions? Soc Sci Med 2014 Aug 20;120C:12-20.  Danchenko N, Rive B, Pendlebury S, Abetz L. Assessing health-related quality of life (HRQOL) in major depressive disorder (MDD): Do commonly used instruments meet EMA requirements for psychometric properties and content relevance. Value in Health 2013 May 2013;16(3):A64.  Danquah FV, Wasserman J, Meininger J, Bergstrom N. Quality of life measures for patients on hemodialysis: a review of psychometric properties. Nephrol Nurs J 2010 May-Jun;37(3):255-69; quiz 270.  Doctor JN, Bleichrodt H, Lin HJ. Health utility bias: a systematic review and meta-analytic evaluation. Med Decis Making 2010 Jan-Feb;30(1):58-67.  Gerhards SAH, Huibers MJH, Theunissen KATM, De Graaf LE, Widdershoven GAM, Evers SMAA. The responsiveness of quality of life utilities to change in depression: A comparison of instruments (SF-6D, EQ-5D, and DFD). Value in Health 2011 July-August 2011;14(5):732-739.  Golomb BA, Vickrey BG, Hays RD. A review of health-related quality-of-life measures in stroke. Pharmacoeconomics 2001;19(2):155-185.  Heintz,E., Wirehn,A.B., Peebo,B.B., Rosenqvist,U., Levin,L.A. QALY weights for diabetic retinopathy--a comparison of health state valuations with HUI-3, EQ-5D, EQ-VAS, and TTO. Value In Health ;15:475.  Hutter BO, Wurtemberger G. Functional capacity (dyspnea) and quality of life in patients with chronic obstructive lung disease (COPD): instruments of assessment and methodological aspects. Pneumologie 1999 Mar;53(3):133-142.  Janssens L, Gorter JW, Ketelaar M, Kramer WL, Holtslag HR. Health-related quality-of-life measures for long-term follow-up in children after major trauma. Qual Life Res 2008 Jun;17(5):701-713.  Johansson P, Agnebrink M, Dahlstrom U, Brostrom A. Measurement of health-related quality of life in chronic heart failure, from a nursing perspective-a review of the literature. Eur J Cardiovasc Nurs 2004 Apr;3(1):7-20.  Johnsen,L.G., Hellum,C., Nygaard,O.P., Storheim,K., Brox,J.I., Rossvoll,I., Leivseth,G., Grotle,M. Comparison of the SF6D, the EQ5D, and the oswestry disability index in patients with chronic low back pain and degenerative disc disease. BMC Musculoskeletal Disorders ;14:148.  Jones GL, Hall JM, Balen AH, Ledger WL. Health-related quality of life measurement in women with polycystic ovary syndrome: a systematic review. Hum Reprod Update 2008 Jan-Feb;14(1):15-25.  Koivunen K, Sintonen H, Lukkarinen H. Properties of the 15D and the Nottingham Health Profile questionnaires in patients with lower limb atherosclerotic disease. Int J Technol Assess Health Care 2007 Summer;23(3):385-391.  Kontodimopoulos N, Aletras VH, Paliouras D, Niakas D. Mapping the cancer-specific EORTC QLQ-C30 to the preference-based EQ-5D, SF-6D, and 15D instruments. Value in Health 2009 November-December 2009;12(8):1151-1157.  Lee EH, Kim CJ, Cho SY, Chae HJ, Lee S, Kim EJ. Monitoring the use of health-related quality of life measurements in Korean studies of patients with diabetes. J Korean Acad Nurs 2011 Aug;41(4):558-567.  Lee,W.J., Song,K.H., Noh,J.H., Choi,Y.J., Jo,M.W. Health-related quality of life using the EuroQol 5D questionnaire in Korean patients with type 2 diabetes. J Korean Med Sci ;27:255.  Luscombe FA. Health-related quality of life measurement in type 2 diabetes. Value Health 2000 Nov-Dec;3 Suppl 1:15-28.  Millier A, Clay E, Chauhan D, Toumi M. Health-related quality of life patient-reported outcomes in schizophrenia. Value in Health 2012 November 2012;15(7):A342.  Ortiz Z, Shea B, Garcia Dieguez M, Boers M, Tugwell P, Boonen A, et al. The responsiveness of generic quality of life instruments in rheumatic diseases. A systematic review of randomized controlled trials. J Rheumatol 1999 Jan;26(1):210-216.  Papaioannou D, Peasgood T, Brazier J, Parry G. The validity of the EQ-5D, SF-6D, SF-36 and SF-12 in mental health conditions: A systematic review. Value in Health 2011 November 2011;14(7):A239.  Patel KK, Veenstra DL, Patrick DL. A review of selected patient-generated outcome measures and their application in clinical trials. Value Health 2003 Sep-Oct;6(5):595-603.  Reaney MD, Martin C, Speight J. Understanding and Assessing the Impact of Alcoholism on Quality of Life: A Systematic Review of the Content Validity of Instruments Used to Assess Health-Related Quality of Life in Alcoholism. Patient 2008 Jul 1;1(3):151-163.  Rowen,D., Young,T., Brazier,J., Gaugris,S. Comparison of generic, condition-specific, and mapped health state utility values for multiple myeloma cancer. Value In Health ;15:1059.  Schmier JK, Halpern MT, Higashi MK, Bakst A. The quality of life impact of acute exacerbations of chronic bronchitis (AECB): a literature review. Qual Life Res 2005 Mar;14(2):329-347.  Scholzel-Dorenbos CJ, van der Steen MJ, Engels LK, Olde Rikkert MG. Assessment of quality of life as outcome in dementia and MCI intervention trials: a systematic review. Alzheimer Dis Assoc Disord 2007 Apr-Jun;21(2):172-178.  Shaw JW, Joish VN, Coons SJ. Onychomycosis: health-related quality of life considerations. Pharmacoeconomics 2002;20(1):23-36.  Smith AB, Cocks K, Taylor M, Parry D. Responsiveness of the EQ-5D in oncology: A meta-analysis. Value in Health 2013 November 2013;16(7):A323-A324.  Soer,R., Reneman,M.F., Speijer,B.L., Coppes,M.H., Vroomen,P.C. Clinimetric properties of the EuroQol-5D in patients with chronic low back pain. Spine Journal ;12:1035.  Street J, Berven S, Fisher C, Ryken T. Health related quality of life assessment in metastatic disease of the spine: a systematic review. Spine (Phila Pa 1976) 2009 Oct 15;34(22 Suppl):S128-34.  Takemoto MLS, Fernandes RA, Cukier FN, Cruz RB, Takemoto MMS, Santos PML, et al. The measurement and valuation of health status using EQ-5D in Brazil: A systematic review. Value in Health 2011 November 2011;14(7):A404.  Teckle,P, Peacock,S, McTaggart-Cowan,H, van,der Hoek. The ability of cancer-specific and generic preference-based instruments to discriminate across clinical and self-reported measures of cancer severities. Health and Quality of Life Outcomes ;9:106.  Tordrup D, Mossman J, Kanavos P. Responsiveness of the EQ-5D to clinical change: is the patient experience adequately represented? Int J Technol Assess Health Care 2014 Jan;30(1):10-19.  Tordrup D, Mossman J, Kanavos P. Shortcomings of EQ-5D in a value-based pricing framework. Value in Health 2013 May 2013;16(3):A10-A11.  Torrecillas IV, Sarabia FN. Arthrosis and quality of life. DOLOR 1999 1999;14(2):121-130.  Turner,N., Campbell,J., Peters,T.J., Wiles,N., Hollinghurst,S. A comparison of four different approaches to measuring health utility in depressed patients. Health and Quality of Life Outcomes ;11:81.  Van Hanswijck de Jonge P, Lloyd A, Horsfall L, Tan R, O'Dwyer PJ. The measurement of chronic pain and health-related quality of life following inguinal hernia repair: a review of the literature. Hernia 2008 Dec;12(6):561-569.  Whitehurst DG, Engel L, Bryan S. Short Form health surveys and related variants in spinal cord injury research: a systematic review. J Spinal Cord Med 2014 Mar;37(2):128-138.  Wilke CT, Pickard AS. Test-retest reliability of the EQ-5D visual analog scale across populations and conditions. Value in Health 2009 May 2009;12(3):A30. |
